# NLRP3 Inflammasome Inhibition Attenuates Diabetic Kidney Injury via the Suppression of Ferroptosis: Mechanistic Insights from In Vivo and In Vitro Models

**DOI:** 10.3390/ijms27104257

**Published:** 2026-05-10

**Authors:** Zhili Tian, Qinghua Yin, Chenglong Zhou, Xiaochu Wu, Fei Liu, Jun Li

**Affiliations:** 1 The Center of Gerontology and Geriatrics, West China Hospital, Sichuan University, Chengdu 610041, China; tianzhili@stu.scu.edu.cn (Z.T.);; 2 National Clinical Research Center for Geriatrics (WCHSCU), West China Hospital, Sichuan University, Chengdu 610041, China; wuxiaochu2018@wchscu.cn; 3 Department of Nephrology, West China Hospital, Sichuan University, Chengdu 610041, China; yinqinghua123@wchscu.edu.cn; 4 Kidney Research Institute, West China Hospital, Sichuan University, Chengdu 610041, China

**Keywords:** diabetic kidney disease, NLRP3 inflammasome, ferroptosis, inflammatory cytokines, oxidative stress

## Abstract

Diabetic kidney disease (DKD) is a primary cause of end-stage renal disease (ESRD), and while ferroptosis is known to contribute to DKD pathogenesis, the regulatory role of the NLRP3 inflammasome in this process remains elusive. To address this research gap, we explored whether NLRP3 inhibition alleviates DKD by suppressing ferroptosis using streptozotocin-induced diabetic wild-type and NLRP3-knockout C57BL/6 mice, alongside high-glucose-cultured (30 mM) human renal tubular epithelial (HK-2) cells with or without siNLRP3 transfection. Inflammatory cytokines (IL-6, TNF-α, and IL-1β) were measured using an ELISA; oxidative stress markers (CSSG, MDA, GSH, and ROS) and the iron ion content via colorimetric assays; mitochondrial morphology by transmission electron microscopy (TEM); and ferroptosis-related proteins (ACSL4, COX2, and GPX4) through Western blotting. Our findings demonstrate that NLRP3-knockout diabetic mice displayed markedly reduced urinary albumin excretion and serum creatinine levels (*p* < 0.01) compared with wild-type diabetic controls, concurrent with suppressed renal iron overload and ferroptosis, diminished inflammatory cytokine levels, and attenuated oxidative stress. Pathological assessments further revealed ameliorated renal fibrosis and preserved mitochondrial ultrastructure in NLRP3-deficient mice. In vitro, siNLRP3 transfection abrogated high-glucose-induced inflammation, oxidative stress, and ferroptosis in HK-2 cells, effects that were reversed by the ferroptosis inducer erastin (*p* < 0.01). Mechanistically, NLRP3 deficiency was associated with upregulated GPX4 expression and downregulated ACSL4 and COX2 expression. Collectively, these results indicate that inhibition of the NLRP3 inflammasome mitigates DKD progression by suppressing ferroptosis, underscoring its translational potential as a therapeutic target for this condition.

## 1. Introduction

Diabetic kidney disease (DKD), a significant microvascular disease, is the primary cause of end-stage renal disease (ESRD) globally, affecting 20–40% of diabetic patients [1,2]. With the global prevalence of diabetes projected to increase from 537 million in 2021 to 783 million by 2045 [3], the incidence of DKD and its associated ESRD burden continues to increase. Epidemiological data have revealed a 74% increase in DKD cases over the past three decades [4], whereas the incidence of diabetes-related ESRD increased from 22.1% to 31.3% between 2000 and 2015 [5]. Current therapeutic strategies (including glycemic control and renin-angiotensin system blockade) only modestly delay disease progression, thus underscoring the urgent need for interventions targeting the multifactorial pathogenesis of DKD. Emerging evidence implicates oxidative stress and inflammation as interdependent drivers of renal injury. Oxidative stress, characterized by an imbalance between the production of reactive oxygen species (ROS) and the capacity of antioxidant defenses, acts as a key driver of cellular injury and mitochondrial dysfunction, thus creating a permissive microenvironment for inflammatory cascades. These inflammatory pathways, which are amplified by inflammatory cytokines (such as interleukin-1β (IL-1β) and tumor necrosis factor-α (TNF-α)), synergistically exacerbate glomerulosclerosis and tubulointerstitial fibrosis [6,7].

The NLRP3 inflammasome, which is a cytosolic multiprotein complex comprising NLRP3, ASC, and procaspase-1, has emerged as a central mediator of inflammation-driven renal injury in DKD [8,9]. Upon activation by hyperglycemia-induced damage-associated molecular patterns (DAMPs, which include mitochondrial ROS and extracellular ATP), the NLRP3 inflammasome catalyzes the caspase-1-dependent cleavage of pro-IL-1β and pro-IL-18 into bioactive cytokines, thus perpetuating renal inflammation and fibrosis [10]. Mechanistically, hyperglycemia triggers mitochondrial DNA damage and oxidative stress, which primes NLRP3 inflammasome assembly and subsequent IL-1β secretion [11]. Preclinical studies have demonstrated that the genetic ablation of NLRP3 in diabetic mice attenuates albuminuria and glomerulosclerosis [12], whereas pharmacological inhibitors (such as MCC950) suppress inflammasome activation and improve renal function [13]. These findings suggest that targeting NLRP3 could represent a promising therapeutic strategy for treating DKD. Despite these advances, the precise mechanisms by which NLRP3 contributes to DKD remain to be fully elucidated.

Ferroptosis, which is an iron-dependent form of regulated cell death characterized by lipid peroxidation, glutathione (GSH) depletion, and glutathione peroxidase 4 (GPX4) inactivation, has recently been demonstrated to be involved in the development of DKD [14]. In diabetic kidney tissue, chronic hyperglycemia leads to increased iron accumulation and oxidative stress. Moreover, increased iron levels catalyze the production of ROS, thus leading to lipid peroxidation and subsequent cell death [15]. Kidney biopsy samples obtained from patients with DKD revealed that the expression levels of GPX4 were significantly decreased compared to those in the control group, whereas lipid ROS levels were increased [16]. Animal models of diabetes, including streptozotocin (STZ)-induced diabetic mice and db/db mice, have demonstrated that iron content is increased in kidney tissue. Pharmacological inhibitors of ferroptosis, such as ferrostatin-1 (Fer-1), can improve kidney function in DKD mice by reducing lipid peroxidation products and iron content [17]. Despite these promising developments, the detailed mechanisms by which ferroptosis contributes to DKD are not yet fully understood.

Recent studies have suggested that there is crosstalk between NLRP3 inflammasome activation and ferroptosis. In cerebral ischemia–reperfusion and sepsis-associated acute kidney injury models, NLRP3 inhibition has been observed to attenuate tissue damage by suppressing both inflammatory cytokine release and ferroptotic marker expression [18,19]. Similarly, according to in vivo and in vitro studies, the inhibition of NLRP3 can mitigate diabetic myocardial injury by suppressing ferroptosis [20]. Despite these findings, the molecular relationship between the NLRP3 inflammasome and ferroptosis in DKD remains unclear. Therefore, the objective of this study was to investigate whether the interaction between the NLRP3 inflammasome and ferroptosis is related to the pathogenesis of DKD.

## 2. Results

### 2.1. Effects of NLRP3 Knockout on Blood Glucose, Body Weight and Renal Function in Mice

**Figure 1:**
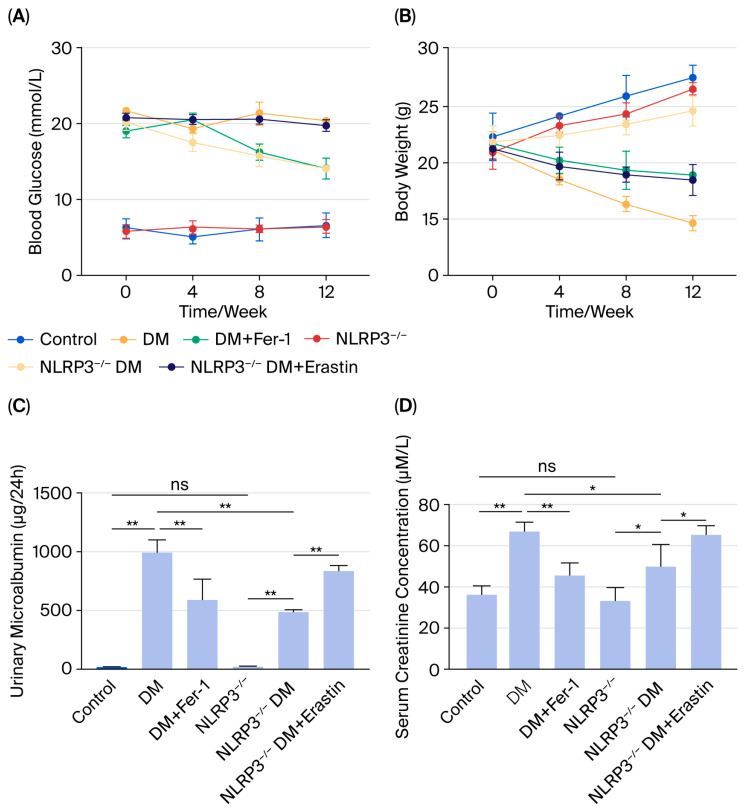
Diabetic kidney injury is alleviated in NLRP3^−/−^ mice. (**A**) Fasting blood glucose levels of the mice treated with or without STZ. In DM and NLRP3^−/−^ DM mice, blood glucose levels were significantly increased and maintained at equal levels throughout the entire 12-week period. (**B**) The body weights of the mice (both with and without STZ treatment) were monitored throughout the 12-week study. (**C**) The urinary albumin levels of the mice over the 12-week period. (**D**) The serum creatinine levels of the mice over the 12-week period. * *p* < 0.05, ** *p* < 0.01, ns = nonsignificant. All of the data are presented as means ± SDs (*n* = 6/8). Abbreviations: DM, diabetes; DM + Fer-1, diabetes + ferrostatin-1; NLRP3^−/−^, NLRP3 knockout; NLRP3^−/−^ DM, NLRP3 knockout + diabetes; NLRP3^−/−^ DM + erastin, NLRP3 knockout + diabetes + erastin.

In this study, all experiments involving NLRP3 knockout (NLRP3^−/−^) mice used six mice per group, while non-NLRP3^−/−^groups used eight mice per group. As shown in Figure 1A,B, the fasting blood glucose levels and body weights of NLRP3^−/−^ wild-type mice did not significantly differ from those of the age-matched littermate controls throughout the 12-week observation period (*p* > 0.05). Subsequent analysis of renal function parameters revealed no significant differences in urinary albumin excretion (Figure 1C) or serum creatinine concentrations (Figure 1D) between the NLRP3^−/−^ and control groups (*p* > 0.05). In the diabetic context, compared with DM control mice, NLRP3^−/−^ DM mice exhibited marked renal protection, with reductions in urinary albumin excretion and serum creatinine levels being observed (both *p* < 0.01). This renoprotective phenotype was recapitulated in DM mice treated with the ferroptosis inhibitor Fer-1. Conversely, administration of the ferroptosis inducer erastin significantly abrogated these benefits in NLRP3^−/−^ DM mice.

### 2.2. NLRP3 Knockout Suppressed Oxidative Stress and Inflammation Levels in Diabetic Mice

**Figure 2:**
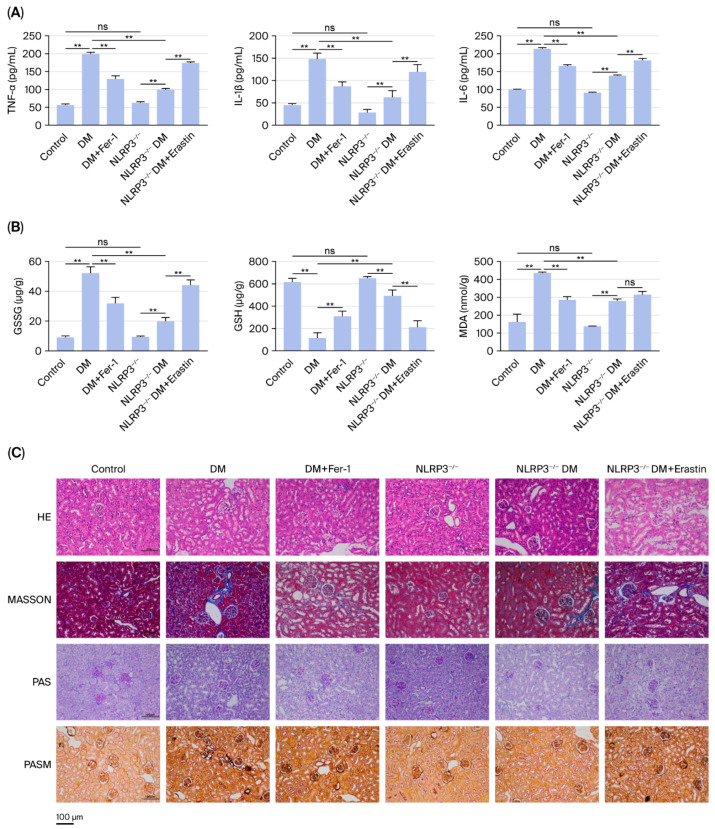
NLRP3 knockout suppressed oxidative stress and inflammation levels in the kidneys of diabetic mice. (**A**) The levels of the inflammatory cytokines IL-6, TNF-α and IL-1β in the kidneys of mice in all groups were examined via assay kits. (**B**) The levels of the oxidative stress factors GSSG, MDA and GSH in the kidneys of mice in all groups were examined via assay kits. (**C**) Representative images of Masson, PAS, and PASM staining of the mouse kidneys. Scale bar = 100 μm. ** *p* < 0.01, ns = nonsignificant. All data are presented as means ± SDs (n = 6/8). Abbreviations: DM, diabetes; DM + Fer-1, diabetes + ferrostatin-1; NLRP3^−/−^, NLRP3 knockout; NLRP3^−/−^ DM, NLRP3 knockout + diabetes; NLRP3^−/−^ DM + erastin, NLRP3 knockout + diabetes + erastin.

We assessed the levels of inflammatory cytokines and lipid peroxidation. Figure 2A,B show that compared with the DM group, the NLRP3^−/−^ DM group demonstrated a significant reduction (*p* < 0.01) in renal inflammation, which was characterized by decreased (*p* < 0.01) levels of inflammatory cytokines (IL-6, TNF-α, and IL-1β), as well as a notable decrease in oxidative stress, evidenced by reduced GSSG and MDA levels and increased GSH levels. Similarly, treatment of DM mice with Fer-1 produced effects akin to those of NLRP3 knockout mice, wherein both inflammation and oxidative stress were significantly decreased in renal tissues. However, in NLRP3^−/−^ DM mice, the administration of erastin significantly reversed the suppression of inflammation and oxidative stress induced by NLRP3 knockout. Furthermore, renal pathological damage was investigated via HE, Masson, PAS and PASM staining. Figure 2C shows that in the DM group, renal tissues exhibited increased ECM accumulation, thickening of the glomerular basement membrane, glycogen deposition, and disordered arrangement of renal tubular cells. In contrast, pathological damage in renal tissues was significantly alleviated in the NLRP3^−/−^ DM group compared with the DM group. Similarly, treatment of DM mice with Fer-1 yielded effects similar to those of NLRP3 knockout mice, wherein ECM accumulation and glycogen deposition were significantly reduced. However, in NLRP3^−/−^ DM mice, the administration of erastin significantly reversed the protective effects of NLRP3 knockout on renal tissue damage.

### 2.3. NLRP3 Knockout Inhibited Ferroptosis in Diabetic Mice

**Figure 3:**
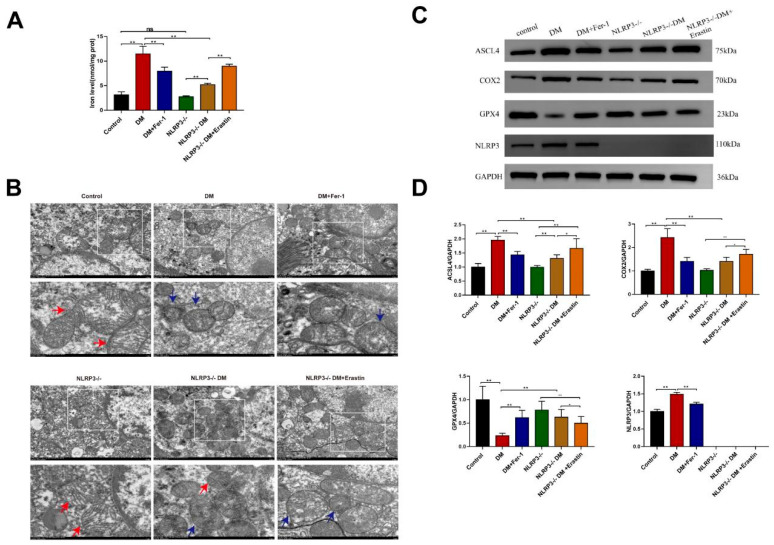
NLRP3 knockout inhibited ferroptosis in the kidneys of diabetic mice. (**A**) Iron ion levels in mouse renal tissues were determined via an assay kit. (**B**) Mitochondria in mouse renal tissues were detected via transmission electron microscopy. Scale bar = 2 μm. The red arrows indicate normal mitochondria, and the blue arrows indicate denatured mitochondria. (**C**) Western blotting analysis of the expression of ACSL4, COX2, GPX4, and NLRP3 in the kidneys mice in all groups. GAPDH served as the reference gene. (**D**) Quantification of the levels of the aforementioned proteins in the kidneys of the mice in all groups. * *p* < 0.05, ** *p* < 0.01, ns = nonsignificant. All data are presented as means ± SDs (n = 6/8). Abbreviations: DM, diabetes; DM + Fer-1, diabetes + ferrostatin-1; NLRP3^−/−^, NLRP3 knockout, NLRP3^−/−^ DM, NLRP3 knockout + diabetes; NLRP3^−/−^ DM + erastin, NLRP3 knockout + diabetes + erastin.

The results shown in Figure 3A reveal that the concentrations of iron ions in the renal tissues of the NLRP3^−/−^ DM group were significantly lower than those in the DM group (*p* < 0.01). Similarly, treatment of DM mice with Fer-1 significantly reduced iron ion levels in renal tissues. In addition, compared with the effects observed in NLRP3^−/−^ DM mice, the administration of erastin markedly reversed the reduction in the iron ion concentration caused by NLRP3 knockout. Transmission electron microscopy (Figure 3B) revealed that in the DM group, renal tissues exhibited a loss of mitochondrial cristae and mitochondrial disorders. However, in the NLRP3^−/−^ DM group, mitochondrial damage was significantly alleviated, with the cristae structure being notably restored compared with that in the DM group. Similarly, treatment with Fer-1 in DM mice significantly reduced mitochondrial damage. In contrast, the administration of erastin in NLRP3^−/−^ DM mice significantly reversed the protective effects of NLRP3 knockout on mitochondrial integrity. Furthermore, we used Western blotting to examine the expression of ferroptosis-related markers. Figure 3C,D show that the upregulated expression of ACSL4 and COX2, as well as the downregulated expression of GPX4, in the DM group were significantly reversed (*p* < 0.05) following NLRP3 knockout in DM mice. Treatment with Fer-1 yielded the same effect, wherein ferroptosis levels in the renal tissues were significantly suppressed (*p* < 0.05). However, compared with the NLRP3^−/−^ DM group, treatment with erastin increased ACSL4 and COX2 expression but decreased GPX4 expression, thereby promoting ferroptosis.

### 2.4. Knockdown of NLRP3 Inhibited Inflammation and Oxidative Stress Levels Induced by High Glucose in HK-2 Cells

**Figure 4:**
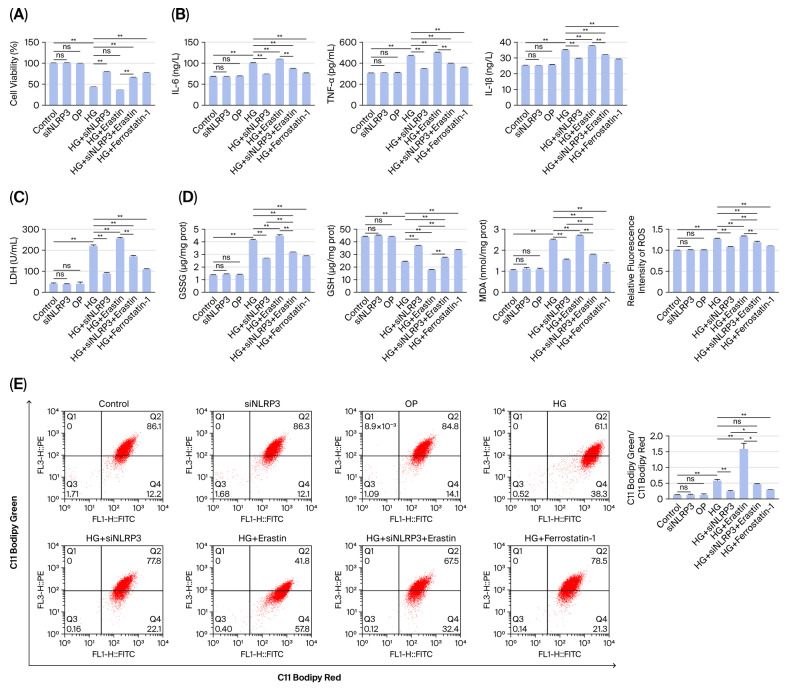
Knockdown of NLRP3 inhibited inflammation and oxidative stress levels induced by high glucose in HK-2 cells. (**A**) Cell viability was determined via a CCK-8 assay. (**B**) The levels of the inflammatory cytokines IL-6, TNF-α and IL-1β were measured via the corresponding assay kits. (**C**) LDH activity was detected in the cell supernatant. (**D**) The levels of the oxidative stress factors GSSG, MDA, GSH and ROS were determined via assay kits. (**E**) Lipid peroxidation was detected in HK-2 cells via C11-BODIPY. * *p* < 0.05, ** *p* < 0.01, ns = nonsignificant. All data are presented as means ± SDs (n = 3). Abbreviations: HG, high glucose; OP, osmotic pressure; HG + siNLRP3, high glucose + siNLRP3; HG + erastin, high glucose + erastin; HG + siNLRP3 + erastin, high glucose + siNLRP3 + erastin; HG + ferrostatin-1, high glucose + ferrostatin-1.

In vitro experiments were conducted on HK-2 cells exposed to high-glucose (HG) conditions. NLRP3 knockdown resulted in no significant difference (*p* > 0.05) in cell viability compared with that of the control group (Figure 4A). However, HG treatment induced marked cellular dysfunction, including significantly reduced cell viability (*p* < 0.01 vs. control; Figure 4A); upregulated expression of the inflammatory cytokines IL-6, TNF-α and IL-1β (all *p* < 0.01 vs. control; Figure 4B); elevated LDH release (*p* < 0.01 vs. control; Figure 4C); and increased oxidative stress, which was characterized by increased GSSG, MDA, and ROS levels (all *p* < 0.01 vs. control), accompanied by GSH depletion (*p* < 0.01 vs. control; Figure 4D). HG also promoted lipid peroxidation, as measured via flow cytometry (*p* < 0.01 vs. control; Figure 4E). Osmotic control with mannitol treatment had no significant effect on these parameters. Notably, NLRP3 knockdown in HG-treated cells (HG + siNLRP3) significantly attenuated HG-induced damage, including improved cell viability (*p* < 0.01 vs. HG), reduced cytokine secretion (all *p* < 0.01 vs. HG), decreased oxidative stress markers (all *p* < 0.01 vs. HG), and suppressed lipid peroxidation (*p* < 0.01 vs. HG). Similarly, treatment with the ferroptosis inhibitor Fer-1 in HG-treated HK-2 cells produced effects comparable to those of NLRP3 knockdown. Furthermore, the ferroptosis inducer erastin partially reversed the protective effects of NLRP3 knockdown in HG + siNLRP3 cells (*p* < 0.05 for viability and oxidative stress markers vs. HG + siNLRP3), thus suggesting an interplay between NLRP3 inflammasome activation and ferroptotic pathways in HG-mediated renal cell injury.

### 2.5. Knockdown of NLRP3 Attenuated the Excessive Activation of Ferroptosis in Cultured HK-2 Cells Under High-Glucose Conditions

**Figure 5:**
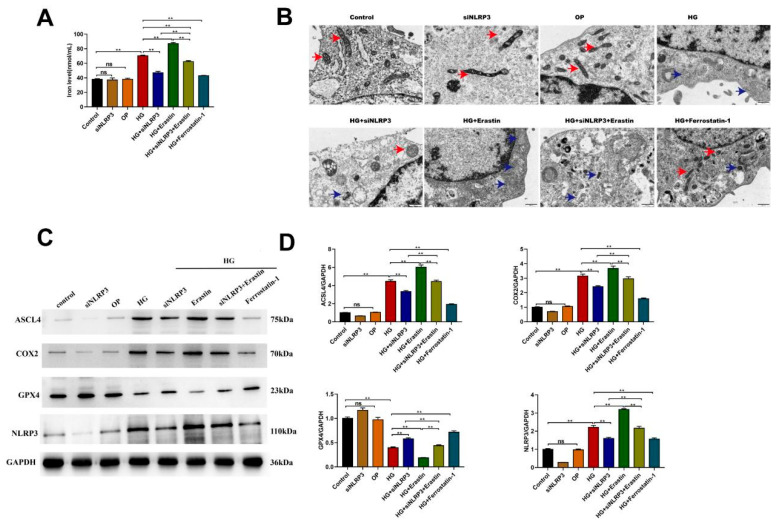
Knockdown of NLRP3 attenuated high-glucose-induced ferroptosis in HK-2 cells. (**A**) The levels of iron ions in HK-2 cells were determined via an assay kit. (**B**) Mitochondria in HK-2 cells were investigated via transmission electron microscopy. Scale bar = 500 nm. The red arrows indicate normal mitochondria, while the blue arrows indicate denatured mitochondria. (**C**) Western blotting analysis of the expression of ACSL4, COX2, GPX4 and NLRP3 in HK-2 cells. (**D**) Quantification of the levels of the aforementioned proteins in HK-2 cells. ** *p* < 0.01, ns = nonsignificant. All data are presented as means ± SDs (n = 3). Abbreviations: HG, high glucose; OP, osmotic pressure; HG + siNLRP3: high glucose + siNLRP3; HG + erastin, high glucose + erastin; HG + siNLRP3 + erastin, high glucose + siNLRP3 + erastin; HG + ferrostatin-1, high glucose + ferrostatin-1.

We subsequently assessed the iron ion concentration, mitochondrial ultrastructure, and ferroptosis marker expression across the experimental groups. In control cells, NLRP3 knockdown had no significant effect on the intracellular iron levels or mitochondrial crista morphology (Figure 5A,B). However, HG treatment induced significant ferroptosis in HK-2 cells, as evidenced by increased iron ion concentrations (*p* < 0.01 vs. the control; Figure 5A), aggravated mitochondrial damage (Figure 5B), upregulated expression of the ferroptosis-related markers ACSL4 and COX2, and downregulated GPX4 expression (all *p* < 0.01 vs. the control; Figure 5C,D). These changes were independent of osmotic stress as mannitol treatment did not affect any of the parameters (all *p* > 0.05 vs. the control). Notably, NLRP3 knockdown attenuated HG-induced ferroptosis in HK-2 cells, reducing iron ion levels (*p* < 0.01 vs. HG; Figure 5A), alleviating mitochondrial damage (Figure 5B), decreasing ASCL4 and COX2 expression, and increasing GPX4 expression (all *p* < 0.01 vs. HG; Figure 5C,D). Similarly, treatment with Fer-1 also reduced HG-induced ferroptosis in HK-2 cells. Conversely, erastin administration partially reversed the protective effects of NLRP3 knockdown against HG-induced ferroptosis in HK-2 cells.

## 3. Discussion

DKD, which is a leading cause of end-stage renal disease, remains a substantial clinical challenge despite therapeutic advancements [21]. Conventional interventions primarily target hemodynamic and metabolic dysregulation, but emerging evidence underscores the pathogenic contributions of dysregulated cell death modalities, particularly with respect to ferroptosis, which is an iron-dependent cell death pathway characterized by lipid peroxidation and the loss of mitochondrial cristae [14,22]. The present study establishes the NLRP3 inflammasome as a critical upstream regulator of ferroptosis in the pathogenesis of DKD, thereby revealing a potential therapeutic axis that connects inflammatory signaling with ferroptotic cell death pathways.

A previous study demonstrated that the ferroptosis biomarkers GPX4, ACSL4 and transferrin (TF) were significantly correlated with the severity of proteinuria, which is a hallmark feature of DKD [23]. Elevated levels of ACSL4, MDA, and ROS are associated with more severe DKD, whereas GPX4 levels are inversely correlated with disease severity, thus underscoring the potential of these markers in predicting and monitoring DKD progression [24]. Wang et al. reported that hyperglycemia can induce oxidative stress, which activates the NLRP3 inflammasome in DKD, thereby intensifying oxidative stress and lipid peroxidation and leading to ferroptosis [25]. These findings indicate that ferroptosis contributes to DKD progression by increasing oxidative stress and lipid peroxidation. Li et al. demonstrated that the Nrf2 signaling pathway, which plays a crucial role in regulating oxidative stress responses, is implicated in ferroptosis associated with DKD. Furthermore, they reported that the upregulation of Nrf2 via fenofibrate therapy can effectively inhibit ferroptosis and slow the progression of DKD in murine models [26]. In this study, we demonstrated that diabetes-induced kidney damage increased both the accumulation of iron ions and the expression levels of the proteins ACSL4 and COX2 while decreasing GPX4 expression. This kidney damage disrupts the normal physiological structure of mitochondria, causing the loss of mitochondrial cristae and overall mitochondrial dysfunction. We also discovered that the use of ferroptosis inhibitors (such as ferrostatin-1) significantly alleviated a range of kidney injury phenotypes induced by diabetes. These features include downregulated expression of inflammatory factors (including IL-6, TNF-α, and IL-1β), the suppression of oxidative stress levels, a reduction in ferroptosis (including decreases in ASCL4 and COX2 expression and increases in GPX4 expression), and alterations in the mitochondrial microstructure. These results collectively demonstrate that ferroptosis plays a critical role in the development and progression of DKD.

The NLRP3 inflammasome functions as a critical regulatory node integrating inflammatory signaling pathways with ferroptotic cell death cascades. Although prior studies have established the role of NLRP3 in regulating ferroptosis in cardiovascular and neurological disorders [27,28], our study revealed a previously underexplored mechanism in diabetic kidney injury. Specifically, diabetes-induced kidney injury upregulates NLRP3 expression in renal tissues, thereby establishing this inflammasome as a critical nexus linking hyperglycemia-induced inflammatory responses to ferroptotic injury. In vivo, compared with DM mice, NLRP3 knockout (NLRP3^−/−^ DM) mice exhibited reduced renal iron deposition and lipid peroxidation products; decreased ACSL4 and COX2 expression; and restored GPX4 activity. Complementary in vitro experiments in HK-2 cells further corroborated this pathway. Specifically, NLRP3 knockdown (via siRNA) or ferrostatin-1 treatment mitigated high-glucose-induced decreases in cell viability, reduced LDH leakage and lipid peroxidation and attenuated inflammation and oxidative stress. Notably, the ferroptosis inducer erastin partially reversed the attenuation of ferroptosis markers observed in NLRP3-silenced HK-2 cells, thus confirming the specificity of the regulatory role of NLRP3 in this ferroptotic pathway.

Notably, this study has several limitations. First, the in vivo experiment in this study were conducted using STZ-induced diabetic mice, which are widely used and reliable models for diabetic research, but they do not capture the full complexity and chronic progression of human DKD. Second, strict randomization grouping was not fully implemented during animal grouping and intervention. Third, in vitro experiments were only performed using HK-2 cells, though DKD is a progressive renal disorder involving multiple renal cell populations, including podocytes, mesangial cells, glomerular endothelial cells and tubular epithelial cells. The use of only one cell type is insufficient to reflect the complexity of the renal microenvironment during DKD progression. Lastly, genetic inhibition of NLRP3 is likely to induce compensatory and adaptive alterations both in vivo and in vitro, which may restrict the direct translational extrapolation of our genetic intervention findings to clinical pharmacological application of NLRP3 inhibitors.

## 4. Materials and Methods

### 4.1. Mouse Model of Diabetes and Treatment

Male C57/BL6 mice (6 weeks old) were procured from the Cavens Experimental Animal Center (Changzhou, China). The animals were housed in a comfortable environment maintained at 23 °C with a natural light/dark cycle of 12 h, and were provided access to water and a regular diet. All experimental animals were “age-matched”, and their feeding environment, circadian rhythm, diet composition, and housing conditions were unified across groups throughout the whole experiment. The diabetic (DM) group received intraperitoneal injections of STZ (8883-66-4, Solarbio, Beijing, China) at a dosage of 50 mg/kg for 5 consecutive days. Fasting blood glucose (FBG) levels were measured 3 days later. Diabetic mice were defined as those with FBG levels >16.7 mmol/L for three consecutive days [29]. The control group (WT) received an equivalent dose of citrate solution via injection. For the ferrostatin-1 treatment, ferrostatin-1 (T6500, TargetMOI, Boston, MA, USA) was administered to diabetic mice beginning at 2 weeks after diabetes induction, with intraperitoneal injections of 1 mg/kg being administered every other day for a total of 10 weeks. NLRP3 knockout (NLRP3^−/−^) mice generated on a C57/BL6 background were obtained from Jackson Laboratory (strain #021302, Bar Harbor, ME, USA). NLRP3^−/−^ diabetic mice (NLRP3 ^−/−^ DM) were generated following the process described for the DM group, whereas the control NLRP3^−/−^ group received an equivalent dose of citrate solution. For erastin treatment, NLRP3^−/−^ DM mice were intraperitoneally injected with 30 mg/kg of erastin (T1765, TargetMOI, Boston, MA, USA) every other day for 10 weeks. The blood glucose levels and weights of the mice were monitored every week. All animals were subsequently euthanized via the CO_2_ euthanasia technique, after which blood, urine, and kidney samples were collected for further analysis. The urine protein and serum creatinine levels were measured using kits from Nanjing Jiancheng Biological Company(Nanjing, China) according to the provided guidelines. The study protocol was approved by the Animal Experimentation Ethics Committee at Sichuan University. All animal care, handling, and experimental procedures were performed in strict accordance with the institutional guidelines and national ethical principles for laboratory animal research.

Removed kidney tissues were preserved in paraffin and optimal cutting temperature (OCT) compound for section preparation. Subsequently, these sections were stained using various techniques, including hematoxylin and eosin (HE), Masson’s trichrome, periodic acid-Schiff (PAS), and periodic acid-silver methenamine (PASM) staining procedures. To perform HE staining, the paraffin sections were first treated with xylene to remove wax and subsequently rehydrated via a series of ethanol solutions. The sections were then stained with hematoxylin (G1120, Solarbio, Beijing, China) for 3 min, rinsed, differentiated in acid alcohol, treated with a bluing reagent, and stained with eosin for 10 min. Paraffin sections were stained with Weigert’s iron hematoxylin for Masson staining, after which they were treated with Biebrich scarlet-acid fuchsin, differentiated in phosphomolybdic-phosphotungstic acid, and finally stained with aniline blue. The sections were dehydrated, treated with xylene for clarification, and then affixed with neutral resin. For PAS staining, the sections were oxidized in periodic acid, rinsed, and subsequently treated with Schiff reagent (G1281, Solarbio, Beijing, China). After being washed in flowing water, the sections were counterstained with hematoxylin, dehydrated in varying concentrations of ethanol, cleared with xylene, and subsequently mounted with neutral resin. For PASM staining, the sections were oxidized in periodic acid, followed by silver methenamine staining (RL3330, Bioroyee, Beijing, China). After rinsing in distilled water, the sections were stained with nuclear fast red, dehydrated with various concentrations of ethanol, cleared with xylene, and subsequently mounted with neutral resin. All sections were analyzed via a light microscope (IX73, Leica, Wetzlar, Germany).

### 4.2. Cell Culture and Treatment

Human renal tubular epithelial (HK-2) cells were acquired from Meisen CTCC Co., Ltd. (Hangzhou, China). The cells were cultured in Dulbecco’s modified Eagle’s medium (DMEM, SH30023.01, Hyclone, Logan, UT, USA) supplemented with 10% fetal bovine serum (FBS, A511-001, Lonsera, Shanghai, China) and 1% penicillin-streptomycin at 37 °C with 5% CO_2_. The cells in the control group were incubated in DMEM with a glucose concentration of 5.6 mM. As an osmotic control (OP), 19.5 mM of mannitol was introduced with 5.6 mM of glucose for 48 h. The cells in the high-glucose (HG) group were treated with DMEM supplemented with 30 mM of glucose (G8150, Solarbio, Beijing, China). Erastin (5 μM, T1765, TargetMOI, Boston, MA, USA) and ferrostatin-1 (1 μM, T6500, TargetMOI, Boston, MA, USA) were used to treat HK-2 cells for 48 h.

### 4.3. siRNA Construction and Transfection

Genecfps Biotech (Jiangsu, China) designed and synthesized the siRNAs targeting NLRP3, and their sequences are presented in Table S1. After the cells were incubated in medium without serum for 24 h, they were transfected with NLRP3 siRNA via Lipofectamine 2000 (11668030, Invitrogen, Carlsbad, CA, USA), and transfection efficiency was determined via RT-PCR analysis (Figure S1). NLRP3 siRNA3 was chosen for subsequent experiments because it demonstrated the highest knockdown efficiency.

### 4.4. CCK-8 Assay

Cell viability was evaluated using the Cell Counting Kit-8 (CCK-8, C0037, Beyotime, Shanghai, China) according to the manufacturer’s protocol. Approximately 1.0 × 10^4^ HK-2 cells/well were seeded in a 96-well plate. After the designated treatment, 10 μL of CCK-8 solution was added to each well, and the cells were incubated at 37 °C for 2 h. Absorbance at 450 nm was subsequently measured via a spectrophotometer (Thermo MK3, Thermo Fisher Scientific, Waltham, MA, USA).

### 4.5. Determination of Oxidative Stress Levels

The levels of malondialdehyde (MDA, BC0025), glutathione (GSH, BC1175), and glutathione disulfide (GSSG, BC1185) in HK-2 cells were determined using specific assay kits purchased from Solarbio (Beijing, China), following the provided guidelines. ROS levels were detected via an ROS assay kit (CA1410, Solarbio, Beijing, China). HK-2 cells were cultured, seeded in 12-well plates, and then stained with 2′,7′-dichlorodihydrofluorescein diacetate (DCFH-DA) at 37 °C for 30 min. Following three washes with phosphate-buffered saline (PBS), fluorescence intensity was measured via fluorescence microscopy. Mouse kidney tissues were rinsed with PBS, homogenized in lysis buffer, and subsequently sonicated. Following sonication, the lysed tissue homogenate was centrifuged at 10,000× *g* for 10 min to eliminate cell debris, and the supernatant was collected. The levels of GSH, GSSG, and MDA in the supernatant were quantified via the appropriate assay kits.

### 4.6. Lactate Dehydrogenase (LDH) Activity Measurement

A total of 2 × 10^6^ cells were harvested and lysed using ice-cold assay buffer. After centrifugation at 10,000× *g* for 15 min, the supernatants were collected for further assays. LDH activity was subsequently assessed via a colorimetric LDH activity assay kit (BC0680, Solarbio, Beijing, China). A 10 µL aliquot of the sample was mixed with 40 µL of assay buffer and 50 µL of reaction mixture in a 96-well plate. After incubation at 33 °C for 15 min, LDH activity was determined by measuring absorbance at 450 nm.

### 4.7. Detection of Inflammatory Cytokines

Supernatants from HK-2 cells and mouse renal tissues in each experimental group were analyzed for inflammatory factors, including TNF-α, IL-1β and interleukin-6 (IL-6). Following standard sample processing procedures, the optical density (OD) at 450 nm was measured via a spectrophotometer (Multiskan MK3, Thermo Fisher Scientific, Waltham, MA, USA). ELISA kits specific for TNF-α (EK282/4-96), IL-1β (EK201B/3-96), and IL-6 (EK206/3-96) were procured from Multi Sciences (Hangzhou, China).

### 4.8. Determination of Lipid Peroxidation

To assess lipid peroxidation, the cells were exposed to 200 nM C11-BODIPY (2362424, Invitrogen, Carlsbad, CA, USA) in HBSS at 37 °C for 30 min. Afterward, the cells were assessed via a BD Accuri C6 flow cytometer equipped with a 488 nm laser.

### 4.9. Detection of Iron Ion Content

An iron assay kit (ab83366, Abcam, Shanghai, China) was used to measure the iron ion concentration. After brief homogenization in iron assay buffer at a ratio of 4–10 volumes, the samples were centrifuged at 16,000× *g* for 10 min to eliminate insoluble substances. The supernatants were then transferred to a 96-well plate and adjusted to a volume of 100 μL with assay buffer. After incubation for 30 min at 37 °C, 100 μL of iron probe was introduced, followed by another 60 min incubation period. The absorbance was measured at 593 nm via a colorimetric microplate reader.

### 4.10. Transmission Electron Microscopy

HK-2 cells and renal tissues were prepared for transmission electron microscopy (TEM) examination. The samples were briefly fixed with 2.5% glutaraldehyde at room temperature for 1 h, followed by postfixation with 1% osmium tetroxide. The samples were subsequently dehydrated in a graded ethanol series at room temperature. After resin penetration, the samples were embedded in epoxy resin and polymerized at 60 °C for 48 h. Ultrathin sections were cut from the embedded samples and stained with uranyl acetate and lead citrate. Images were captured using an HT7700 transmission electron microscope (Hitachi, Tokyo, Japan).

### 4.11. Western Blotting

The tissue or cell samples were completely mixed in a glass blender and subsequently treated with 400 μL of RIPA lysis buffer for 30 min. Afterward the cell extracts were centrifuged at 13,000 rpm for 10 min at 4 °C. The resulting protein supernatants were then utilized for protein quantification by using the improved BCA Assay Kit (BL521A, Biosharp, Heifei, China). Equal amounts of protein (∼30 μg per sample) were used for SDS-PAGE. After being transferred to PVDF membranes, the blots were blocked with a 5% (*w*/*v*) nonfat dried milk solution containing 5% BSA at 37 °C for 2 h. Subsequently, the membranes were incubated overnight at 4 °C with primary antibodies, including ACSL4 (1:5000, 81196-1-RR, Proteintech, Wuhan, China), COX2 (1:2000, 66351-1-IG, Proteintech, Wuhan, China), GPX4 (1:1000, 67763-1-IG, Proteintech, Wuhan, China), NLRP3 (1:2000, A5652, ABclonal, Wuhan, China), and GAPDH (1:50,000, 60004-1-Ig, Proteintech, Wuhan, China). Afterward, the blots were exposed to secondary antibodies conjugated with HRP at room temperature for 4 h. Signals were detected by using ECL reagents (ECL-0011, Dingguo, Beijing, China) and captured with a FluorChem imaging system (ChemiScope 5300 Pro, CLINX, Shanghai, China).

### 4.12. Statistical Analysis

The data are expressed as means ± standard deviations (SDs). Prior to formal statistical analysis, the normality assumption was verified using the Shapiro–Wilk test, and homogeneity of variances was assessed using Levene’s test to ensure the validity of parametric test application. Statistical analysis was conducted via GraphPad Prism 7.0 software (GraphPad Software, San Diego, CA, USA). A one-way analysis of variance (ANOVA) was employed, followed by Tukey’s honest significant difference (HSD) post hoc test, for multiple comparisons between groups. For data that failed to meet the normality or homogeneity of variance assumptions, the non-parametric Kruskal–Wallis H test followed by Dunn’s post hoc test with Bonferroni correction was used instead. *p* values less than 0.05 were considered statistically significant. All experiments were repeated at least three times (n  =  3) for each experimental group, and independent replicates were used for statistical analysis.

## 5. Conclusions

The present study demonstrates that ferroptosis plays a critical pathogenic role in the development and progression of DKD. The NLRP3 inflammasome emerged as a key upstream regulator of ferroptosis in DKD, thereby suggesting the potential existence of a regulatory axis that links inflammatory signaling to ferroptotic cell death in diabetes-induced renal injury. NLRP3 is a promising therapeutic target for the clinical management of DKD, as its inhibition alleviates DKD progression by suppressing ferroptosis, We acknowledge this study’s limitations (STZ mouse model, single cell type (HK-2), and potential genetic NLRP3 inhibition compensatory effects), guiding future research to validate the translational potential of NLRP3-targeted therapy.

## Data Availability

The data is contained within the article and Supplementary Materials.

## Supplementary Materials

The following supporting information can be downloaded at: https://www.mdpi.com/article/10.3390/ijms27104257/s1.

## Abbreviations

The following abbreviations are used in this manuscript:

NLRP3	NOD-Like Receptor Family Pyrin Domain Containing 3
IL-6	Interleukin-6
TNF-α	Tumor Necrosis Factor-α
IL-1β	Interleukin-1β
ELISA	Enzyme-Linked Immunosorbent Assay
GSSG	Glutathione Disulfide
MDA	Malondialdehyde
ACSL4	Acyl-CoA Synthetase Long-Chain Family Member 4
COX2	Cyclooxygenase-2
RIPA	RadioImmunoPrecipitation Assay Buffer
SDS-PAGE	Sodium Dodecyl Sulfate-PolyAcrylamide Gel Electrophoresis
PVDF	Polyvinylidene Fluoride
BSA	Bovine Serum Albumin
ECL	Enhanced ChemiLuminescence
