# Supplementary material for: NLRP3 Inflammasome Inhibition Attenuates Diabetic Kidney Injury via the Suppression of Ferroptosis: Mechanistic Insights from In Vivo and In Vitro Models
Source: Int J Mol Sci. 2026 May 10;27(10):4257. doi: 10.3390/ijms27104257 (PMC13207044; doi:10.3390/ijms27104257)
Supplement: Supplementary file 1 [file ijms-27-04257-s001.zip › ijms-4253576-supplementary.pdf]

Table S1 Sequences of three candidate NLRP3 siRNAs for screening and validation

Primer	Sense Strand	Antisense Strand
NLRP3-1	CCGUAAGAAGUACAGAAAGUATT	UACUUUCUGUACUUCUUACGGTT
NLRP3-2	CCAGCCAGAGUCUAACUGAAUTT	AUUCAGUUAGACUCUGGCUGGTT
NLRP3-3	CCACAUGACUUUCCAGGAGUUTT	AACUCCUGGAAAGUCAUGUGGTT

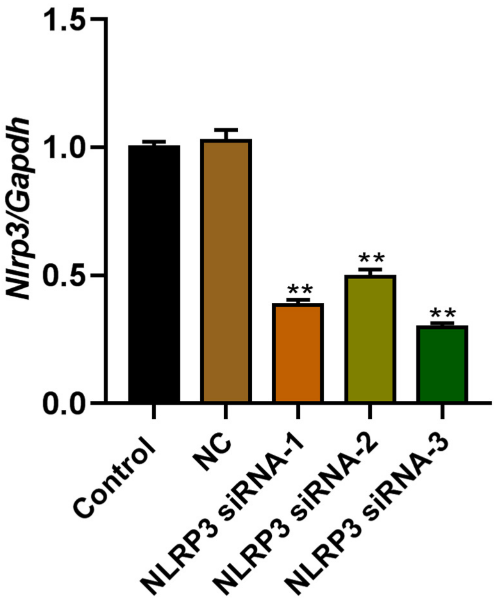

Figure S1. Knockdown efficiency of three NLRP3-specific siRNAs determined by RT-qPCR.

Note: The relative mRNA expression level of Nlrp3 was examined by reverse transcription quantitative real-time polymerase chain reaction (RT-qPCR) in cells transfected with control (NC), NLRP3 siRNA-1, NLRP3 siRNA-2, or NLRP3 siRNA-3. Data are presented as mean ± SEM. **P < 0.01 versus NC group (n = 3).
